# Effects of Ultrasound Treatments on the Technological Characteristics of Restructured Cured Cooked Products Elaborated With Pale, Soft and Exudative Pork Loins

**DOI:** 10.1111/1750-3841.71581

**Published:** 2026-10-18

**Authors:** Amanda Camilo Graciano, Miriam de Andrade Pereira, Larissa Marçal dos Santos, Marielle Maria de Oliveira Paula, Douglas Roberto Guimarães Silva, Alcinéia de Lemos Souza Ramos, Eduardo Mendes Ramos

**Affiliations:** ^1^ Department of Food Science, School of Agricultural Sciences of Lavras Federal University of Lavras (UFLA) Lavras Minas Gerais Brazil; ^2^ President Tancredo de Almeida Neves University Center (UNIPTAN) São João Del Rei Minas Gerais Brazil

**Keywords:** color indices, sonication, texture profile, water‐holding capacity

## Abstract

The present study evaluated the effects of high‐intensity ultrasound treatment (acoustic intensities of 192 or 301 W/cm^2^ at 20 KHz for up to 15 min) on the technological properties of restructured cured cooked products manufactured with pork loins from different quality classes: reddish‐pink, firm, and non‐exudative (RFN) and pale, soft, and exudative (PSE). Meat quality class was the main factor affecting product characteristics. Products manufactured with RFN loin had (*p* < 0.05) higher moisture content (76.08% vs. 75.37%) and pH values (6.30 vs. 6.12) and reduced (*p* < 0.05) cooking (4.34% vs. 6.28%) and reheating (4.74% vs. 6.23%) losses. In contrast, PSE‐based products had (*p* < 0.05) lower values of all texture attributes evaluated (hardness, cohesiveness, springiness, adhesiveness, and chewiness) and lower (*p* < 0.05) residual nitrite content (26.72 vs. 52.26 mg/kg). The use of PSE loins resulted (*p* < 0.05) in products with a lighter (L* 61.19 vs. 59.04) and a more yellowish–pinkish hue (*h* 55.80° vs. 53.22°) color than with RFN loins. Lipid oxidation was not affected (*p* > 0.05) by ultrasound treatment or meat quality class. Sonication time influenced (*p* < 0.05) only the texture profile, with treatments applied for 10 min resulting in lower hardness and chewiness values regardless of ultrasound power. Overall, the results indicate that the technological quality of restructured cooked pork loins was primarily influenced by raw material quality rather than ultrasound application. Under the evaluated conditions, ultrasound had limited effects on product functionality and was not sufficient to overcome the technological limitations associated with the use of PSE meat.

**Practical Applications:**

This study indicates that selecting pork based on its quality class is more effective than applying high‐intensity ultrasound to improve the quality of restructured cooked cured products. Under the conditions evaluated, ultrasound had limited effects on product functionality and did not overcome the processing disadvantages associated with PSE meat. The adoption of raw material quality classification programs may help processors achieve more consistent product quality and reduce losses during manufacturing and subsequent product use.

## 1 Introduction

Pork processing is one of the most important sectors of the global food industry. Among the wide variety of processed pork products, cured meats stand out as highly popular products. These products are formulated with curing salts (nitrite/nitrate) and subjected to thermal processing, resulting in mildly flavored ready‐to‐eat products that are commonly consumed cold in sandwiches and snacks. In Brazil, in addition to classification based on ingredient and additive composition, these products are categorized according to the raw material used during processing as “Presunto cozido” (cooked ham), produced from sectioned cuts of pork ham; “Apresuntado” (restructured cooked ham), manufactured from ground pork ham and/or pork shoulder meat; and “Fiambres” (lunch meats), prepared from ground meat obtained from any part of the pork carcass or from other animal species (Brasil 2022a, 2022b; 2023a). More recently, the Brazilian meat industry has introduced fiambre‐type products with technological and sensory characteristics like those of cooked ham and restructured cooked ham but manufactured using pork loin and/or pork tenderloin cuts. These products offer a stronger nutritional appeal due to the naturally lower fat content of these cuts.

In the production of cooked cured meat products, one of the most important factors affecting both product quality and the economic efficiency of processing is the quality of the raw material used (Toldrá et al. 2010). Over recent decades, intensive genetic selection for leaner pigs, driven by consumer demand, has resulted in an increased incidence of abnormal pork meat. One of the most significant and frequently observed quality defects in pork is the pale, soft, and exudative (PSE) condition, which contrasts with the reddish‐pink color, firm texture, and non‐exudative (RFN) meat considered normal and suitable for processing (Ramos and Gomide 2017; Torres Filho et al. 2018). According to Cazedey et al. (2016), the incidence of PSE pork in Brazilian slaughterhouses may reach up to 40%, leading to its frequent utilization in processed meat products. The most valuable cuts of the carcass, particularly the ham and loin, are also the most affected by the PSE condition (Prändl et al. 1994).

Because PSE meat compromises protein functionality, its use in cooked cured products is associated with several technological defects, including pale color, low water‐holding capacity (WHC), and consequently, reduced cooking yield, as well as granular, brittle texture and poor overall consistency (O'Neill et al. 2003; Paula et al. 2019; Haddad et al. 2018). In addition to lowering industrial yield, the loss of water and soluble components from cured products represents an important technological, sensory, and economic issue. Excessive purge accumulation inside packaging negatively affects consumer perception, whereas moisture losses during slicing after removal from primary packaging may also result in economic losses for retailers. Furthermore, cooked cured products are widely used as ingredients in frozen ready‐to‐eat meals, such as pizzas and pasta dishes. In these applications, thawing and reheating losses (RHL) may cause additional problems, including texture deterioration of both the meat product and the accompanying food matrix, as well as excessive exudation in the final prepared dish (Pedroso and Demiate 2008; Dutra et al. 2012). Therefore, pre‐classification of meat based on technological quality serves as a valuable tool for the industry, enabling the appropriate allocation of raw material to various applications and minimizing negative impacts on the yield, texture, WHC, and color characteristics of processed products. However, Brazilian slaughterhouses do not routinely classify carcasses according to meat quality. Consequently, the implementation of strategies for the identification and management of PSE and RFN meat could improve process standardization and enhance the consistency and overall quality of processed meat products.

Beyond the selection and targeting of raw materials, the use of technologies capable of enhancing product characteristics has been investigated as a strategy to minimize technological limitations and meet the demand for efficient, sustainable processes. In this context, nonthermal technologies have attracted growing interest because they enable food preservation under relatively low‐temperature conditions; this helps preserve heat‐sensitive characteristics and overcomes the limitations of traditional methods, thereby ensuring food safety, quality, and sustainability (Ağagündüz et al. 2025). Among these nonthermal technologies, ultrasound has gained increasing attention due to its potential to improve processing yield and key quality attributes, including texture, color, and flavor of meat products. Ultrasound is an innovative technology based on the application of sound waves at frequencies ranging from 20 kHz to 10 MHz and can be classified into two frequency ranges: low‐intensity ultrasound, which operates at high frequencies between 100 kHz and 10 MHz, and high‐intensity ultrasound, which uses lower frequencies between 20 and 100 kHz (Firouz et al. 2022). High‐intensity ultrasound has been extensively investigated because of its cavitation effects and mechanical action, which may enhance mass transfer, salt diffusion, and microstructural modifications, while also improving yield and texture in certain applications, particularly in reduced‐sodium formulations or products with technological limitations (Li et al. 2024; Guo et al. 2024). Barretto et al. (2018, 2020) reported that sonication reduced the total fluid loss during cooking, increased instrumental hardness, and improved consumer acceptance of salty taste in restructured cooked cured hams.

Although recent reviews have indicated that ultrasound may contribute to improvements in WHC and structural properties of meat products, its effects are highly dependent on operational conditions such as power/intensity, treatment time, temperature, stage of application, and product formulation (Wang et al. 2025; Kang et al. 2021). Therefore, the present study aimed to evaluate the effects of ultrasonic processing parameters (time and power) on the technological characteristics of restructured cooked products manufactured from pork loins of different quality classes (PSE and RFN), in order to determine whether this technology can mitigate the effects of raw material variability on the technological performance of the final product.

## 2 Materials and Methods

### 2.1 Materials

Pork loins (M. *Longissimus lumborum*) were obtained from a commercial slaughterhouse and meat processing plant (Nutrili Alimentos Ltda., Lavras, MG, Brazil) operating under Federal Inspection Service supervision. Twenty‐four hours postmortem, loins from chilled carcasses were deboned, packaged, identified, stored with ice in insulated containers, and transported to the Meat and Meat Products Technology Laboratory (LabCarnes) of the Department of Food Science (DCA) at the Federal University of Lavras (UFLA), with an average transport time of 30 min.

In the LabCarnes, the loins were evaluated for drip loss (DL) and lightness (L*) and classified according to meat quality criteria established by Torres Filho et al. (2018): PSE, L* > 52 and DL > 6%; and RFN, L* = 44–52 and DL = 2%–6%. Eight loins classified as PSE (L* = 55.96 ± 1.35 and DL = 7.49% ± 1.04%) and eight classified as RFN (L* = 51.56 ± 1.30 and DL = 3.46% ± 1.25%) were selected, vacuum‐packaged (BS420; R. Baião, Ubá, MG, Brazil), and frozen (at −18°C) for subsequent use in the experiment.

### 2.2 Restructured Pork Loins Batter Manufacture

The fiambres were produced on the basis of a commercial formulation commonly used for “Apresuntados” products, which typically include extender ingredients such as cassava starch, carrageenan, and soy protein isolate (SPI). These extenders have been reported to mitigate the adverse effects associated with the use of PSE meat in cooked meat products, as demonstrated by Motzer et al. (1998) and Schilling et al. (2004). The basic formulation consisted of 64% pork loin, 30% water (4°C), 1.8% salt, 1.4% SPI (New Max Industrial Ltda., Americana, SP, Brazil), 4% cassava starch (Amafil Indústria e Comércio Ltda., São Lourenço, PR, Brazil), 0.25% sodium tripolyphosphate (Ibrac Aditivos e Condimentos, Rio Claro, SP, Brazil), 0.20% carrageenan (New Max Industrial Ltda., Americana, SP, Brazil), 0.20% monosodium glutamate (Ajinomoto, São Paulo, SP, Brazil), 0.20% California‐style seasoning (New Max Industrial Ltda., Americana, SP, Brazil), 0.10% sodium erythorbate (Ibrac Aditivos e Condimentos, Rio Claro, SP, Brazil), and 0.02% sodium nitrite (Vetec Química Ltda., Rio de Janeiro, RJ, Brazil). Production was carried out in four independent batches (true replicates), performed on different days.

For each meat quality class (PSE and RFN), two pairs of loins were randomly selected for processing. The loins were thawed at 4°C for 24 h while still vacuum‐packaged to ensure uniform thawing and minimize exudate losses. Subsequently the packages were opened, visible fat was removed, and the lean meat was ground using a kidney plate disc in an industrial grinder (Beccaro Ltda., Rio Claro, SP, Brazil). The formulation ingredients were incorporated into the meat batter and mixed in a vacuum tumbler (RM20, R. Baião Indústria e Comércio, Ubá, MG, Brazil) for 15 min to ensure complete extraction and solubilization of myofibrillar proteins as well as uniform distribution of additives. After mixing, the meat batters were vacuum‐packaged (BS420, R. Baião, Ubá, MG, Brazil) in seven portions of 500 g each and stored (at 4°C for about 30 min) prior to the sonication process.

### 2.3 Ultrasound Treatments and Cooking

The ultrasonic treatments were defined to evaluate the main factors potentially influencing the quality of cured meat products, namely, ultrasound power (255 and 400 W) and treatment time (5, 10, and 15 min). The treatments were performed using a high‐intensity ultrasound processor (Eco‐Sonics QR850; Ultronique, Indaiatuba, SP, Brazil) equipped with a 13‐mm titanium probe (macro tip) operating at a frequency of 20 kHz. The nominal energy parameters of ultrasound treatments were summarized in Table 1.

**TABLE 1:** Nominal energy parameters^a^ of ultrasound treatments applied to cooked cured products.

Ultrasound power (W)	AI (W/cm^2^)	PD (W/cm^3^)	Treatment time (min)	SE (J/g)	ED (J/cm^3^)
255	192	0.319	5	153	95.6
255	192	0.319	10	306	191.3
255	192	0.319	15	459	286.9
400	301	0.500	5	240	150.0
400	301	0.500	10	480	300.0
400	301	0.500	15	720	450.0

Abbreviations: AI, acoustic intensity; ED, energy density; PD, power density; SE, specific energy.

^a^ Determined according to Mason and Lorimer (2002), Bai et al. (2023), and Moura et al. (2024).

The experimental design is illustrated in Figure 1. For each batch of meat quality class, the seven vacuum‐packaged meat batters were randomly assigned to seven sonication treatments according to ultrasound power and treatment time: T1 = control; T2 = 255 W/5 min; T3 = 255 W/10 min; T4 = 255 W/15 min; T5 = 400 W/5 min; T6 = 400 W/10 min; and T7 = 400 W/15 min. The sonication process was conducted indirectly in a stainless‐steel vessel (18 cm diameter × 13.5 cm height) containing approximately 800 mL of cold water (at 4°C) to prevent excessive temperature increase during treatment. The vacuum‐packaged meat batter was placed at the bottom of the vessel, whereas the ultrasonic probe was immersed centrally, maintaining 5 cm between the probe tip and the sample. Water temperature was monitored before and after each treatment using a digital thermometer and remained between 7°C and 8°C at the end of each sonication process, indicating that the treatment was conducted under low‐temperature conditions.

**FIGURE 1:**
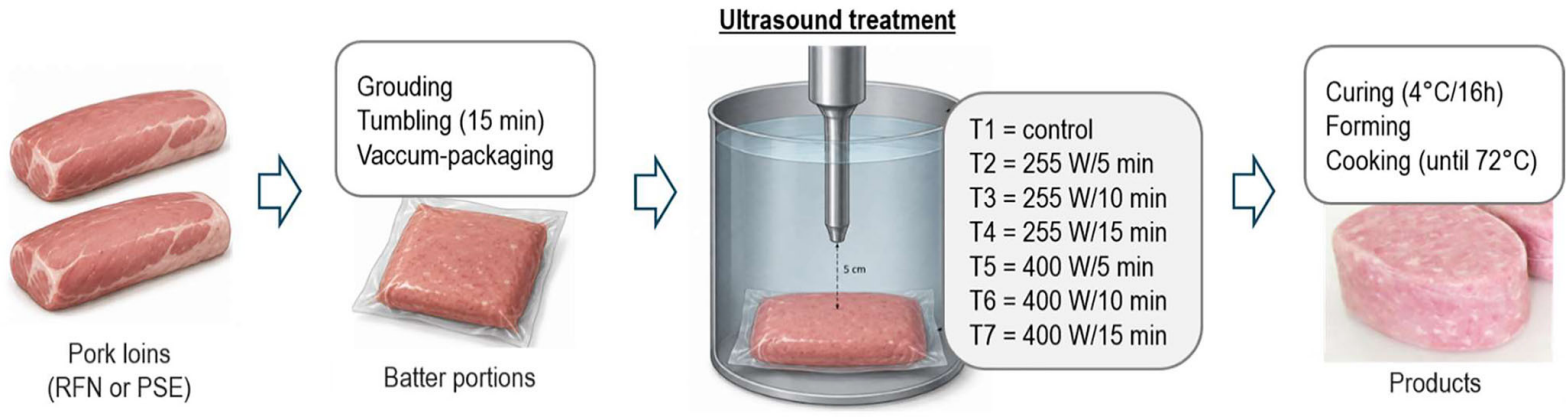
Experimental design for the loin batters of each pork quality class: pale, soft, and exudative (PSE) and reddish‐pink, firm, and non‐exudative (RFN) conditions.

After sonication, the meat batters were transferred to metallic molds and held under refrigeration (4°C) for 16 h for curing process. Subsequently, the products were cooked in a water bath (at 80°C) up to an internal temperature of 72°C. After cooking, the products were immediately cooled in an ice‐water bath and stored under refrigeration (4°C) for 24 h prior to analysis.

### 2.4 Analytical Methods

#### 2.4.1 Chemical Composition and pH Analysis

The pH values of the products were measured using a portable pH meter (HI99.163; Hanna Instruments Brasil, São Paulo, SP, Brazil) previously calibrated with standard buffer solutions at pH 4.1 and 7.0. Measurements were performed in three distinct regions of each product.

Proximate composition analyses were performed in triplicate according to the methodologies described by the Association of Official Analytical Chemists (AOAC 2012). Moisture content was determined by direct drying of the samples in a forced‐air oven at 105°C until constant weight was achieved (method 925.45b). Ether extract content was determined using a Soxhlet extraction system with petroleum ether as the solvent (method 920.39). Protein content was calculated on the basis of nitrogen levels determined by the Kjeldahl method, using a conversion factor of 6.25 (method 960.52). Ash content was determined by incineration of the samples in a muffle furnace at 550°C until complete carbonization (method 923.03). Total carbohydrate content was calculated by difference, subtracting the sum of moisture, fat, protein, and ash percentages from the total percentage.

#### 2.4.2 WHC Evaluation

The effects of the treatments on the WHC of the products were evaluated under different stress conditions, including cooking loss (CL), expressible moisture (EM), storage under negative pressure (syneresis), freeze–thaw loss (FTL), and RHL, based on adaptations of the methodologies described by Haddad et al. (2018).

The cooked products were unpacked, dried with absorbent paper, and weighed to determine CL, expressed as the percentage of mass lost during cooking. Then subsamples were obtained for the remaining analyses.

For syneresis, 10 cubes (1 cm edge) were weighed together, vacuum‐packaged, and stored under refrigeration (4°C) for 10 days. Packaged samples were subjected to thermal stress conditions (∼20°C for 2 h) every 2 days. After each period, the cubes were removed from the packaging, gently dried with absorbent paper, and reweighed. The percentage of mass loss was calculated relative to the initial weight.

EM was determined using five cylindrical samples (25 mm diameter × 25 mm height) obtained with a cylindrical cutter. Samples were weighed and subjected to the texture profile analysis (TPA) test using a TA.XT2i texture analyzer (Stable Micro Systems Inc., UK). After the test (two compression cycles), samples were reweighed, and the percentage of mass loss was calculated relative to the initial sample weight before TPA test.

For RHL analysis, four rectangular segments (10 × 10 mm^2^ cross‐section × 30 mm length) from each sample were individually weighed and immersed in 300 mL of boiling water for 6 min. The segments were then drained on paper towels, cooled at 4°C for 6 min, and reweighed to calculate the percentage of mass loss.

FTL was evaluated using three rectangular samples (25 × 25 mm^2^ cross‐section × 10 mm thickness), which were individually weighed, packaged in plastic bags, and frozen at −18°C. After 24 h of freezing, the three pieces from each treatment were thawed at room temperature for 4 h. One sample was separated after the first cycle (Cycle 1), whereas the remaining two samples were refrozen. This procedure was repeated twice more to obtain samples corresponding to Cycles 2 and 3. In each cycle, the thawed sample was unpackaged, wrapped in filter paper, placed between two glass plates, and compressed with a standardized 5.0‐kg weight for 5 min. After compression, the sample was removed from the filter paper and reweighed, and the percentage of mass loss was calculated for each freeze–thaw cycle.

#### 2.4.3 Texture Profile Analysis

The TPA was performed using a TA.XT2i texture analyzer (Stable Micro Systems Inc., UK) according to the procedure described by Ramos and Gomide (2017). Five cylindrical samples (25 mm diameter × 25 mm height) from EM analysis were individually compressed twice between two sheets of filter paper using a 12.5‐cm diameter compression plate until reaching 60% of its original height. No resting time was allowed between the two compression cycles. The force–time deformation curve was recorded at a compression speed of 200 mm/min, from which the following texture parameters were determined: hardness (N), cohesiveness, adhesiveness (N × mm), springiness, and chewiness (N).

#### 2.4.4 Instrumental Color

Instrumental color was evaluated using a CM‐5 spectrophotometer (Konica Minolta Inc., Tokyo, Japan) with a 30‐mm aperture, specular component excluded (SCE), D65 illuminant, and 10° observer angle. Samples were cut in half, and the reflectance spectrum (400–700 nm), as well as the color indices lightness (L*), chroma (C*), and hue angle (*h*, in degrees), were obtained from the average of five readings taken at different positions on the internal surface.

The cured color index, associated with nitrosylhemochrome pigment formation, was determined using the reflectance ratio at wavelengths 650 and 570 nm (R650/570 nm) according to King et al. (2023).

#### 2.4.5 Lipid Oxidation

Lipid oxidation was evaluated using the thiobarbituric acid reactive substances (TBARS) method, according to Andrade et al. (2024). Approximately 5 g of sample were homogenized (Turratec TE 102; TECNAL, Piracicaba, SP, Brazil) with 15 mL of extraction solution containing 3.62% perchloric acid and 0.01% butylated hydroxytoluene (BHT), followed by filtration. A 1‐mL aliquot of the filtrate was mixed with 1 mL of thiobarbituric acid solution (0.02 M in 3.86% perchloric acid) and incubated in a water bath at 90°C for exactly 30 min. After cooling in an ice bath for 5 min, absorbance was measured at 532 nm. TBARS values were expressed as milligrams of malondialdehyde (MDA) per kilogram of product (mg MDA/kg), using a standard calibration curve prepared with 1,1,3,3‐tetraethoxypropane (TEP).

#### 2.4.6 Residual Nitrite

Residual nitrite content (NO_2_R) was determined according to the method described by Andrade et al. (2024). Approximately 4 g of homogenized sample was added to 40 mL of distilled water preheated to 80°C and incubated in a water bath at 80°C for 2 h. The mixture was then filtered and diluted to a final volume of 250 mL with distilled water. A 1‐mL aliquot was used for the Griess reaction. First, 0.2 mL of 1% sulfanilamide solution (prepared in 0.5 M HCl) was added, and the mixture was incubated at room temperature in the dark for 5 min. Subsequently, 0.2 mL of 0.1% *N*‐(1‐naphthyl)ethylenediamine dihydrochloride (NED) solution was added, followed by incubation for an additional 15 min. Absorbance of the final mixture was measured at 540 nm, and residual nitrite content (mg/kg) was quantified using a sodium nitrite standard calibration curve.

### 2.5 Statistical Analysis

The experiment was conducted in a completely randomized design (CRD), with a split‐plot 2 × 2 × 4 factorial arrangement, consisting of two meat quality classes (PSE and RFN) in the main plot and two ultrasonic power levels (250 and 400 W) and four sonication times (0, 5, 10, and 15 min) in the subplot. Four genuine batches (replicates) were conducted, totaling 64 experimental units. Data were subjected to analysis of variance (ANOVA), and when significant effects were observed, means were compared using Tukey's test at a significance level of 5%. Statistical analyses were performed using Statistica 5.0 software (StatSoft, Poland).

## 3 Results and Discussion

### 3.1 Chemical Composition

The chemical composition results demonstrated that only meat quality class significantly affected (*p* < 0.05) the evaluated parameters, whereas ultrasound power and treatment time had no significant (*p* > 0.05) influence on the analyzed variables (Table 2). Products manufactured with RFN meat had (*p* > 0.05) higher moisture content and lower fat content compared with those produced using PSE meat. Despite these differences, protein, ash, and carbohydrate contents did not differ significantly (*p* > 0.05) between RFN and PSE products. Similar findings were reported by Neves et al. (2020), who observed that meat quality primarily affected technological attributes related to water retention, with limited impact on protein and mineral contents.

**TABLE 2:** Effects of pork meat quality class (*C*) and ultrasound (US) power (*P*) and sonication time (*T*) on the proximate composition of cooked cured products.

Effects	Source of variation	Moisture (%)	Protein (%)	Ether extract (%)	Ash (%)	Carbohydrates (%)
Pork class, *C*	RFN	76.08^x^	16.68	1.86^x^	3.05	2.35
	PSE	75.37^y^	16.90	2.03^y^	3.23	2.47
US power	255	75.73	16.72	1.93	3.12	2.51
*P* (W)	400	75.63	16.85	1.97	3.07	2.48
US time	0	75.42	16.78	2.17	3.11	2.52
*T* (min)	5	75.71	16.82	1.82	3.12	2.53
	10	75.98	16.65	1.78	3.11	2.48
	15	75.62	16.87	2.03	3.03	2.45
	SEM	0.12	0.08	0.10	0.05	0.06
Pr > *F* ^a^	*C*	**0.032**	0.308	**0.003**	0.079	0.575
	*P*	0.835	0.451	0.939	0.375	0.535
	*T*	0.675	0.818	0.552	0.697	0.719
	*C* × *P*	0.930	0.980	0.853	0.871	0.888
	*C* × *T*	0.965	0.864	0.484	0.609	0.596
	*P* × *T*	0.996	0.772	0.960	0.202	0.291
	*C* × *P* × *T*	0.721	0.999	0.656	0.766	0.597

*Note*: Means followed by different letters (^x,y^) between pork classes differ (*p* < 0.05) by the *F* test.

Abbreviations: PSE, pale, soft, and exudative loin; RFN, reddish‐pink, firm, and non‐exudative loin; SEM, standard error of the mean (*n* = 64).

^a^ Significant values (*p* < 0.05) were represented in bold.

The application of ultrasound at the evaluated power levels (255 and 400 W) did not promote significant changes in the proximate composition of the products. This is in accordance with the observation of Barretto et al. (2018) that the main effects of ultrasound are associated with modifications in the microstructural and functional properties of the meat matrix rather than alterations in the proximate composition of the final product.

### 3.2 pH and Mass Losses

Only meat quality class significantly affected (*p* > 0.05) the pH values, and mass losses parameters (Table 3). Product pH was higher (*p* > 0.05) in RFN meat products compared with those produced from PSE meat. This difference may be attributed to the more intense postmortem acidification process at a high muscle temperature, typically observed in PSE meat as a consequence of accelerated glycolysis (Kim et al. 2014). This condition, particularly during the first hour postmortem, compromises protein functionality, and consequently, the WHC of meat products (Huff‐Lonergan and Lonergan 2005; Schäfer et al. 2002), which also explains the greater CL, syneresis, and RHL observed in products manufactured with PSE meat. These higher losses found in PSE‐based products indicate that the protein matrix formed after cooking was structurally less stable, resulting in increased water losses during both cooking and reheating, regardless of ultrasound application. On the other hand, EM and FTL were not affected (*p* > 0.05) by any of the evaluated factors.

**TABLE 3:** Effects of pork meat quality class (*C*) and ultrasound (US) power (*P*) and sonication time (*T*) on pH values and mass losses of cooked cured products.

							FTL (cycles)		
Effects	Source of variation	pH	CL	SYN	EM	RHL	1	2	3
Pork class, *C*	RFN	6.30^x^	4.34^x^	5.75^x^	1.97	4.74^x^	11.18	13.38	15.41
	PSE	6.12^y^	6.28^y^	6.41^y^	1.84	6.23^y^	11.77	13.52	15.49
									
US power	255	6.21	5.40	6.13	1.93	5.39	11.58	13.66	15.63
*P* (W)	400	6.21	5.22	6.03	1.89	5.61	11.36	13.24	15.27
US time	0	6.20	5.17	5.58	2.12	5.05	11.02	12.13	15.59
*T* (min)	5	6.21	5.33	6.00	1.90	5.89	11.49	14.33	15.32
	10	6.22	5.38	6.60	1.86	5.02	11.25	13.65	15.49
	15	6.22	5.37	6.15	1.76	6.00	12.13	13.71	15.40
	SEM	0.02	0.16	0.19	0.10	0.22	0.24	0.38	0.27
Pr > *F* ^a^	*C*	**<0.001**	**<0.001**	**0.047**	0.300	**<0.001**	0.102	0.848	0.824
	*P*	0.889	0.282	0.760	0.734	0.565	0.536	0.555	0.340
	*T*	0.581	0.784	0.251	0.254	0.110	0.155	0.174	0.961
	*C* × *P*	0.562	0.479	0.395	0.331	0.924	0.315	0.860	0.856
	*C* × *T*	0.240	0.473	0.560	0.121	0.472	0.337	0.231	0.115
	*P* × *T*	0.086	0.257	0.970	0.983	0.950	0.604	0.928	0.934
	*C* × *P* × *T*	0.875	0.610	0.445	0.395	0.593	0.879	0.671	0.835

*Note*: Means followed by different letters (^x,y^) between pork classes differ (*p* < 0.05) by the *F* test.

Abbreviations: CL, cooking loss; EM, expressible moisture; FTL, freeze–thaw loss; PSE, pale, soft, and exudative loin; RFN, reddish‐pink, firm, and non‐exudative loin; RHL, reheating loss; SEM, standard error of the mean (*n* = 64); SYN, syneresis.

^a^ Significant values (*p* < 0.05) were represented in bold.

O'Neill et al. (2003) demonstrated that the use of PSE pork in the production of cooked ham significantly compromises the technological quality of the final product. Hams manufactured with PSE meat exhibited significantly greater drip and CLs compared with those produced from normal meat, in addition to reduced slicing ability.

Under the evaluated conditions, ultrasound did not (*p* > 0.05) promote consistent improvements in the technological performance of the products regarding water losses (Table 2). The positive effects of ultrasound are frequently associated with structural modifications induced in myofibrillar proteins, including enhanced protein extraction and conformational changes such as secondary structure transitions (α‐helix → β‐sheet), formation of new disulfide bonds, and development of denser and more homogeneous gel networks, resulting in increased protein–water interactions, greater WHC and reduced CL (Wang et al. 2025; Amiri et al. 2018). It is important to note that sonication in the present study was performed indirectly, with the probe positioned 5 cm above the vacuum‐packaged sample immersed in chilled water. Consequently, the acoustic intensities reported (192 and 301 W/cm^2^) correspond to the emitting surface of the probe and not to the effective intensity reaching the meat batter. Attenuation through the water column, the packaging material, and the batter itself likely resulted in considerably lower intensities at the sample level, which may have contributed to the limited effects observed.

In cooked cured meat products, some studies have reported beneficial effects of ultrasound on WHC. Barretto et al. (2018, 2020) observed lower total fluid release after cooking in low‐sodium restructured cooked hams (0.75% and 0.50% salt, respectively) subjected to ultrasound treatment (20 kHz; 600 W/cm^2^ for 10 min) prior to tumbling and cooking. However, in the present study, the salt concentration used was considerably higher (1.8%), which likely promoted sufficient extraction and solubilization of myofibrillar proteins, thereby mitigating the additional positive effects on these proteins induced by acoustic cavitation. Although the use of PSE meat in processed products may impair protein functionality in a manner similar to the reduced extraction and solubilization caused by salt reduction, Haddad et al. (2018) and Paula et al. (2019) reported that salt reduction in restructured cured products exerts a greater impact on final product quality than the incorporation of PSE meat into the formulation.

An additional factor that should be considered when interpreting the absence of significant ultrasound effects on WHC is the inclusion of extender ingredients (cassava starch, carrageenan, and SPI) in the product formulation. As previously described, these ingredients are widely used in restructured meat products because of their ability to enhance water retention and partially compensate for the technological deficiencies associated with PSE meat (Motzer et al. 1998; Schilling et al. 2004). Nevertheless, even in the presence of these functional ingredients, ultrasound application under the conditions evaluated in the present study was not sufficient to mitigate the negative effects associated with the incorporation of PSE meat into the formulation.

In high‐salt cooked hams (2% salt), Zhang et al. (2024) demonstrated that the application of high‐intensity ultrasound (20 kHz; 300 W for 40 min) during the tumbling process, prior to thermal gelation, enhanced soluble protein extraction and improved the structural cohesion of the product, resulting in greater WHC, and consequently, lower CL. However, the yield improvements associated with sonication were only observed by these authors in samples subjected to tumbling times longer than 4 h. These findings suggest that the effectiveness of ultrasound is highly dependent on the processing conditions employed, being influenced not only by sonication parameters such as intensity, frequency, and treatment time, but also by the extent of protein extraction and solubilization achieved before thermal processing. In the present study, ultrasound was applied after only 15 min of tumbling. Therefore, it can be hypothesized that the static holding period following ultrasound treatment (16 h at 4°C) was not long enough to allow the sonication‐induced changes in protein functionality to produce significant improvements in the WHC of the products.

### 3.3 Texture Profile Analysis

Similarly to the results observed for mass losses, a significant effect of pork meat quality class was found for all evaluated texture attributes (Table 4), confirming that raw material quality was the major factor influencing the final product profile.

**TABLE 4:** Effects of pork meat quality class (*C*) and ultrasound (US) power (*P*) and sonication time (*T*) on texture attributes of cooked cured products.

Effects	Source of variation	Hardness (N)	Adhesiveness (*N* × mm)	Springiness	Cohesiveness	Chewiness (N)
Pork class, *C*	RFN	83.66^x^	0.53^x^	0.78^x^	0.33^x^	376.62^x^
	PSE	66.99^y^	0.30^y^	0.73^y^	0.28^y^	237.94^y^
US power	255	75.25	0.42	0.75	0.30	305.48
*P* (W)	400	75.39	0.42	0.76	0.30	309.07
US time	0	77.73^a^	0.43	0.76	0.31	324.25^a^
*T* (min)	5	75.01^ab^	0.45	0.76	0.30	305.98^ab^
	10	72.19^b^	0.35	0.74	0.30	285.34^b^
	15	76.35^a^	0.45	0.75	0.30	313.53^a^
	SEM	1.28	0.04	0.01	<0.01	10.31
Pr > *F* ^a^	*C*	**<0.001**	**0.002**	**<0.001**	**<0.001**	**<0.001**
	*P*	0.901	0.934	0.451	0.840	0.646
	*T*	**0.011**	0.711	0.847	0.308	**0.008**
	*C* × *P*	0.713	0.937	0.811	0.505	0.435
	*C* × *T*	0.903	0.909	0.772	0.409	0.656
	*P* × *T*	0.853	0.777	0.842	0.402	0.895
	*C* × *P* × *T*	0.268	0.827	0.992	0.103	0.107

*Note*: Means followed by different letters between (^x,y^) pork classes and (^a,b^) US time differ (*p *< 0.05) by the *F* test and Tukey test, respectively.

Abbreviations: PSE, pale, soft, and exudative loin; RFN, reddish‐pink, firm, and non‐exudative loin; SEM, standard error of the mean (*n *= 64).

^a^ Significant values (*p *< 0.05) were represented in bold.

In general, all texture attribute values were reduced when PSE meat was used in the formulation, indicating that the protein matrix formed in the manufactured products with RFN meat produced a firmer product, with greater (*p* < 0.05) adhesiveness, cohesiveness, springiness, and chewiness than those elaborated with PSE meat. This finding is consistent with the lower functionality of myofibrillar proteins in PSE meat due to early protein denaturation, which impairs the formation of a continuous and stable protein network during cooking and consequently reduces product cohesiveness and firmness (Haddad et al. 2018).

In a study evaluating myofibrillar proteins from PSE meat, Wu et al. (2022) demonstrated that adjusting the system with sodium bicarbonate significantly improved gel texture properties, including hardness, springiness, cohesiveness, and chewiness, in addition to increasing cooking yield. Lower values for the texture attributes springiness, cohesiveness, and chewiness were also reported by McDonagh et al. (2005) in cooked hams manufactured from pork muscles visually classified as pale compared with those selected as having “good” color. However, these authors did not observe significant differences in hardness between the samples. Paula et al. (2019) not only failed to observe differences in the hardness of restructured cooked hams manufactured with RFN and PSE meats but also reported greater springiness in products elaborated with PSE meat, whereas Neves et al. (2020) did not observe effects on any texture attributes. It is important to emphasize that these authors used pork ham muscles classified only visually (based on paleness) or associated with carcasses classified according to loin quality, which, despite being the primary muscle used for meat quality prediction, does not accurately define the extent of the PSE condition in ham muscles (Person et al. 2005; Knecht et al. 2018). In this regard, evaluating the effects of different proportions of PSE loin meat in cooked cured products, Haddad et al. (2018) observed a reduction in hardness in products containing higher proportions of PSE meat, which was directly reflected in lower chewiness values. However, these authors did not find significant effects of PSE meat proportion on product adhesiveness, cohesiveness, or springiness.

Changes in the microstructure of the protein gel affect not only instrumental texture, but also the sensory perception of the product, particularly attributes related to juiciness and the perception of cohesiveness during mastication (Paula et al. 2019). At the same time, data reported in the literature indicate that the negative effects associated with the use of PSE meat in cooked products can be minimized through strategies that improve protein functionality during processing, such as higher salt addition and the incorporation of extender ingredients (Haddad et al. 2018; Neves et al. 2020; Schilling et al. 2004; Motzer et al. 1998).

Regarding the effects of ultrasound on texture attributes, only sonication time significantly affected hardness and chewiness (Table 4). Sonication for 10 min, regardless of power level, may have promoted modifications in the protein matrix that resulted in a less firm product, reducing (*p* < 0.05) hardness and chewiness values. Ultrasound may alter the structural organization of myofibrillar proteins through the mechanical effects of acoustic cavitation, promoting partial disruption of protein structures, changes in intermolecular interactions, and reorganization of the meat gel after thermal processing (Wang et al. 2025; Kang et al. 2021).

Barretto et al. (2018, 2020) also observed an increased hardness and chewiness values in ultrasound‐treated low‐sodium restructured cooked hams (600 W/cm^2^ for 10 min). Moreover, while investigating the impact of ultrasound application (20 kHz; 300 W for 40 min) during the tumbling process of cooked ham meat batter, Zhang et al. (2024) also observed a reduction in hardness values regardless of tumbling time (4, 6, or 8 h). Although springiness increased with sonication, chewiness decreased only in samples tumbled for 6 h. The authors attributed these effects to a balance between extraction and solubilization of myofibrillar proteins during tumbling, which promoted the formation of a more cohesive and elastic protein network that became more compact after thermal denaturation, thereby improving the textural characteristics of the final product.

As observed for mass loss analyses, in cooked cured products the effects of ultrasound on texture appear to be less pronounced when compared with emulsified systems, in which acoustic cavitation acts more effectively on protein organization and emulsion stability. Cichoski et al. (2019) reported that ultrasound application in emulsified meat products promoted modifications in the emulsion matrix and positively altered texture profile parameters, improving the technological quality of meat emulsions. Zhou et al. (2021) reported that ultrasound improves emulsifying and rheological properties by reducing droplet size and promoting protein unfolding, thereby increasing protein–water and protein–fat interactions. However, as pointed out before, the direction and magnitude of these effects are strongly dependent on the operational conditions employed, including ultrasound intensity, frequency, exposure time, and the characteristics of the meat matrix.

### 3.4 Lipid Oxidation, Residual Nitrite, and Instrumental Color

TBARS values were not affected (*p* < 0.05) by any of the evaluated factors (Table 5), indicating that neither the use of PSE meat nor sonication, within the power and time ranges applied in the present study, was sufficient to induce lipid oxidation in the freshly cooked cured products. Haddad et al. (2018) reported that the proportion of PSE meat used in the manufacture of restructured smoked‐cured pork loins was one of the main factors affecting lipid oxidation in the products, which was attributed to the fact that lipid oxidation is favored in meats with lower pH values. O'Neill et al. (2003) also observed higher TBARS values in cooked hams produced with PSE meat compared with those manufactured using RFN meat. Ultrasound treatment, particularly under excessive and prolonged exposure conditions, may promote the formation of highly oxidative species and trigger intense lipid and protein oxidation (Guo et al. 2024). However, Kim et al. (2025) and Barretto et al. (2018) likewise did not observe differences in TBARS values between cured cooked products subjected or not subjected to ultrasound treatment.

**TABLE 5:** Effects of pork meat quality class (*C*) and ultrasound (US) power (*P*) and sonication time (*T*) on the TBARS values, residual nitrite (NO_2_R), and color indices of cooked cured products.

Effects	Source of variation	TBARS (mg MAD/kg)	NO_2_R (mg/kg)	L*	C*	*h* (°)	Cured color
Pork class, *C*	RFN	0.47	52.26^x^	59.04^x^	19.25	53.22^x^	2.28^x^
	PSE	0.52	26.72^y^	61.19^y^	20.02	55.80^y^	2.18^y^
US power	255	0.54	39.64	59.89	19.47	55.03	2.22
*P* (W)	400	0.45	39.34	60.31	19.79	53.91	2.23
US time	0	0.53	41.77	59.46	18.27	54.63	2.23
*T* (min)	5	0.45	36.62	59.87	21.08	55.53	2.23
	10	0.44	39.67	60.08	20.09	53.22	2.24
	15	0.56	39.90	60.97	19.12	54.43	2.22
	SEM	0.03	2.47	1.16	1.27	1.14	0.06
Pr > *F* ^a^	*C*	0.362	**<0.001**	**0.046**	0.586	**<0.001**	**0.032**
	*P*	0.106	0.906	0.705	1.000	0.360	0.703
	*T*	0.225	0.536	0.656	0.120	0.590	0.978
	*C* × *P*	0.443	0.966	0.396	1.000	0.449	0.559
	*C* × *T*	0.354	0.726	0.912	0.076	0.738	0.705
	*P* × *T*	0.264	0.944	0.573	0.979	0.331	0.507
	*C* × *P* × *T*	0.501	0.991	0.926	0.875	0.441	0.280

*Note*: Cured color = R650/570 nm index. Means followed by different letters between (^x,y^) pork classes differ (*p* < 0.05) by the *F* test.

Abbreviations: C*, chrome; *h*, hue angle; L*, lightness; MAD, malondialdehyde; PSE, pale, soft, and exudative loin; RFN, reddish‐pink, firm, and non‐exudative loin; SEM, standard error of the mean (*n* = 64); TBARS, thiobarbituric acid reactive substances.

^a^ Significant values (*p* < 0.05) were represented in bold.

Residual nitrite content (NO_2_R) of the products was significantly higher (*p* < 0.05) in those manufactured with RFN meat than with PSE meat (Table 4). The lower NO_2_R values observed in PSE‐based products were likely associated with its lower pH values (Table 3), as the conversion of nitrite into nitric oxide (NO) is favored under acidic conditions (Andrade et al. 2025). Nevertheless, this lower NO_2_R value observed in PSE samples may compromise product color stability during extended storage (not evaluated in this experiment), as a certain amount of residual nitrite is important to provide a continuous source of NO, preventing color fading (Sebranek and Bacus 2007). Furthermore, although lower residual nitrite levels may have implications for microbiological stability during extended refrigerated storage, the markedly lower NO_2_R observed in PSE‐based products (∼49% lower than in RFN products) is consistent with current efforts to reduce residual nitrite in cured meats due to concerns regarding the endogenous formation of N‐nitrosamines during digestion (Andrade et al. 2025; Andrade et al. 2026). However, both PSE‐ and RFN‐based products remained well below the maximum residual nitrite limit of 150 mg/kg established by Brazilian legislation (Brasil 2023b), indicating that the observed differences, while technologically relevant, do not constitute an immediate regulatory concern.

Under the conditions of the experiment, ultrasound treatment did not promote consistent changes in NO_2_R values, which is in agreement with the findings of Kim et al. (2025), who reported that variations in NO_2_R were more strongly related to the chemical environment of the curing system, particularly the presence of ascorbic acid, rather than to the application of ultrasound itself.

Color indices were affected (*p* < 0.05) only by the quality class of the raw material (Table 5). The use of PSE loins in the formulation resulted in products with a lighter (higher L* values) and a more yellowish–pinkish hue (higher *h* values) color than those formulated with RFN meat. In addition, the instrumental cured color indicator (R650/570 nm) was lower (*p* < 0.05) in the PSE samples, although the observed values remained within the range considered indicative of “excellent cured color perception” (2.0–2.6) according to King et al. (2023).

These changes in product color are consistent with the characteristics typically associated with PSE meat, in which the rapid postmortem pH decline causes protein denaturation, increasing light scattering and resulting in a paler appearance of the meat (Kim et al. 2014; Ramos and Gomide 2017). Some studies (Schilling et al. 2004; Motzer et al. 1998; McDonagh et al. 2005; O'Neill et al. 2003) have also reported increased L* values in cooked hams produced with PSE meat compared with normal meat, whereas others (Haddad et al. 2018; Neves et al. 2020; Paula et al. 2019) did not observe significant effects on any color indices.

Finally, the absence of a significant effect of ultrasound application on color indices (Table 5) suggests that, under the evaluated conditions, this technology did not produce perceptible changes in the final appearance of the products. Similar results were reported by Barretto et al. (2020) in reduced‐sodium meat products (0.50% salt), with or without partial replacement of NaCl by KCl, in which ultrasound application did not promote significant differences (*p* < 0.05) in color indices. However, Barretto et al. (2018) observed a reduction in L* values in products containing 0.75% salt without KCl substitution. These findings, combined with those observed in the present experiment, suggest that, in addition to ultrasound‐related parameters (power, time, etc.), the effects of sonication on color indices may also depend on formulation composition, including meat quality, and on the interactions between meat matrix proteins and the various additives used in the product.

## 4 Conclusion

The results of the present study demonstrated that pork meat quality class was the main factor affecting the quality of restructured cooked pork loins, whereas ultrasound application, regardless of the power level and treatment time employed, was not sufficient to overcome the technological limitations associated with PSE meat. The use of PSE meat negatively affected several quality attributes, confirming the detrimental impact of reduced protein functionality associated with this meat condition. Nevertheless, sonication time influenced texture attributes indicating that ultrasound may promote structural modifications in the protein matrix.

From a practical perspective, high‐intensity ultrasound appears to have limited effectiveness in mitigating the technological deficiencies associated with PSE meat in cooked cured products, even in formulations containing conventional salt levels and functional extender ingredients. Therefore, the identification and segregation of pork based on quality class remain essential strategies for minimizing raw material variability and ensuring consistent quality and technological performance in processed meat products.

## Author Contributions

**Amanda Camilo Graciano**: conceptualization, investigation, writing – original draft. **Miriam de Andrade Pereira**: methodology, investigation. **Larissa Marçal dos Santos**: investigation. **Marielle Maria de Oliveira Paula**: validation, writing – review and editing. **Douglas Roberto Guimarães Silva**: writing – review and editing. **Alcinéia de Lemos Souza Ramos**: methodology, formal analysis, writing – review and editing. **Eduardo Mendes Ramos**: conceptualization, methodology, supervision, funding acquisition, project administration, writing – review and editing.

## Conflicts of Interest

The authors declare no conflicts of interest.
